# Editorial: Metabolic flexibility of microbial methane oxidation

**DOI:** 10.3389/fmicb.2022.1079906

**Published:** 2022-11-22

**Authors:** Ruo He, Paul L. E. Bodelier, Zhongjun Jia, Peng Xing

**Affiliations:** ^1^International Science and Technology Cooperation Platform for Low-Carbon Recycling of Waste and Green Development, Zhejiang Gongshang University, Hangzhou, China; ^2^Netherlands Institute of Ecology (NIOO-KNAW), Wageningen, Netherlands; ^3^Institute of Soil Science, Chinese Academy of Sciences, Nanjing, China; ^4^Nanjing Institute of Geography & Limnology, Chinese Academy of Sciences, Nanjing, China

**Keywords:** microbial methane oxidation, nitrogen fixation, methanotrophs, methanethiol oxidation, microbial community

Methane is the second most important greenhouse gas after carbon dioxide and contributes up to 20% to current global warming. Microbial methane oxidation plays a significant role in mitigating methane emission to the atmosphere and therefore is a crucial process to understand in the battle against climate change, where methane mitigation has highest priority as recently reported by the IPCC. Methanotrophs have long been believed to be exclusively dependent on methane for carbon and energy. However, discoveries in the past two decades have demonstrated a much higher metabolic versatility of these environmentally highly relevant microbes. Methanotrophs have been shown to utilize multi-carbon compounds in addition to methane, presumably deriving a competitive advantage from this metabolic versatility. They also can oxidize ammonia, fix nitrogen, catalyze denitrification, execute parts of sulfur metabolism and glycolysis under environmental stress such as nitrate, volatile sulfur compounds and low oxygen concentrations. Additionally, it has been shown that microbial methane oxidation can be coupled to denitrification, sulfate reduction and metal reduction such as iron and manganese under anoxic conditions. These novel insights into the metabolic versatility of methanotrophs also has implication for the interaction with other organisms in the environment. Methanotrophs have been demonstrated to provide methane-derived carbon to food chains in terrestrial and aquatic ecosystems designated them as “primary producers”.

This Research Topic consists of seven original articles aiming at identifying metabolic pathways and biogeochemical processes involved in environmental microbial methane oxidation. Three of these studies investigated the metabolic capabilities of proteobacterial methanotrophs, including methanethiol oxidation, N_2_ fixation, and their interaction with non-methanotrophs in natural habitats. Three studies focused on the characterization of the community structure and metabolic characteristics of methanotrophs in association with archaea under anoxic conditions. The last study reported on the response of microbial communities and methane fluxes to climate change in glacier foreland soils.

Methanotrophs have been shown to be versatile in the metabolization of substrates such as ammonia, multicarbon compounds and nitrate in addition to methane, thereby presumably deriving competitive advantages in their habitats. Schmitz et al. used *Methylacidiphilum fumariolicum* SolV as a model organism and found that methanotrophs could oxidize methanethiol and produce H_2_S, which could be further oxidized. This might be a widespread detoxification mechanism in methanotrophs in a range of environments. Cao et al. reported on methane oxidation-induced N_2_ fixation that led to methanotrophy-primed mineralization, which accounted for 21.7% of total nitrogen involved in facilitation of root carbon turnover. DNA-based stable isotope probing indicated that gammaproteobacterial methanotrophs *(i.e., Methylomona*s) dominated N_2_ fixation in methane-consuming rice roots. Similarly, Cui et al. observed that N_2_ fixation was enhanced in the presence of methane, which might be related with Diazotrophic *Methylosinus* species of the family *Methylocystaceae*. These findings expand the knowledge on interactions of methane and nitrogen cycling providing input for management strategies to mitigate methane emissions from natural and engineering systems while reducing nitrogen input.

Anaerobic oxidation of methane (AOM) plays a crucial role in regulating methane emissions from anaerobic habitats, which has been reported to be make use of electron acceptors such as NO${}_{3}^{-}$, NO${}_{2}^{-}$, SO${}_{4}^{2-}$ and Fe^3+^. Anaerobic methanotrophic archaea (ANME) are the main agents in AOM that can be divided into three groups: ANME-1 (ANME-1a and ANME-1b), ANME-2 (ANME-2a, ANME-2b) and ANME-3. Although ANME are often linked to sulfate reduction, Chen et al. reported the participation of ANME in iron reduction coupled to methane oxidation (Fe-AOM) with ANME-1a representatives as key players in an active cold seep in the mid-Okinawa Trough. Co-occurrence analysis revealed that the methanogen/ANME population and some heterotrophic microbial groups could interact metabolically through an anaerobic food chain. Ouboter et al. demonstrated the capacity of members of the *Candidatus* genus Methanoperedens to transfer electrons extracellularly forming a bio-electrochemical system. These results hold great promise in coupling methane mitigation to the generation of electricity. Additionally, Su et al. also reported that proteobacterial methanotrophs could grow on methane under both oxic and anoxic conditions, although methane oxidation activities were an order of magnitude higher under oxic conditions. This suggested that bacterial AOM by facultative aerobic methane oxidizers might be of much larger environmental significance in reducing methane emissions than previously thought.

Human activities and climate change can influence microbial communities and methane fluxes from the environment. Methanogens and methanotrophs are responsible for biological methane cycling in various environments, and their relative activities determine the global methane dynamics. Next to this, methane dynamics in the natural environments is controlled by environmental factors such as nutrient availability, vegetation coverage, terrain micro-topography and temperature. Xing et al. reported that due to global warming and subsequent glacier retreat, glacier foreland soils changed from methane sinks to methane sources under influence of glacial meltwater. This suggests that enhanced glacial retreat may have positive feedbacks global warming by the modulation of methane cycling microbial communities in these systems.

Taken together, the set of papers in this Research Topic broaden our horizon on the traits of methanotrophs. The expanding versatility may have major implication in the control of methane cycling in many relevant habitats. It emphasizes the role of methanotrophs in carbon, nitrogen and sulfur cycling and the interactions with non-methanotrophs, the outcome of which can have major implication for many biogeochemical cycles. Further understanding of this fascinating group of microbes and their metabolic flexibility will be crucial in not only predict future methane fluxes but also the impact on other elemental cycles. Additionally, knowledge on traits of methanotrophs will be key in developing biotechnological applications of methanotrophs for facilitating a sustainable future.

## Author contributions

RH, PB, ZJ, and PX wrote and edited the manuscript. All authors have contributed substantially to the article and approved the manuscript for publication.

## Conflict of interest

The authors declare that the research was conducted in the absence of any commercial or financial relationships that could be construed as a potential conflict of interest.

## Publisher's note

All claims expressed in this article are solely those of the authors and do not necessarily represent those of their affiliated organizations, or those of the publisher, the editors and the reviewers. Any product that may be evaluated in this article, or claim that may be made by its manufacturer, is not guaranteed or endorsed by the publisher.

